# Druggable Mendelian randomization prioritizes CDH2 and supports finerenone as a candidate therapeutic strategy for diabetic retinopathy

**DOI:** 10.3389/fphar.2026.1865172

**Published:** 2026-07-15

**Authors:** Xiangyang Liu, Ning Zhang, Shi Wu, Man Zhang, Bei Sun, Liming Chen

**Affiliations:** 1 NHC Key Laboratory of Hormones and Development and Tianjin Key Laboratory of Metabolic Diseases, Tianjin Medical University Chu Hsien-I Memorial Hospital & Institute of Endocrinology, Tianjin, China; 2 Department of Endocrinology, 983 Hospital of the Joint Logistics Support Force of the People’s Liberation Army of China, Tianjin, China

**Keywords:** CDH2, diabetic retinopathy, drug repurposing, finerenone, Mendelian randomization, N-cadherin, oxygen-induced retinopathy, target prioritization

## Abstract

Diabetic retinopathy remains a major cause of vision loss, and therapeutic strategies beyond anti-vascular endothelial growth factor treatment are still needed. This study aimed to identify genetically supported druggable targets for diabetic retinopathy and to evaluate finerenone as a candidate therapeutic intervention in experimental retinopathy. We performed druggable Mendelian randomization by integrating druggable-gene resources, blood cis-expression quantitative trait locus data, and a large genome-wide association dataset for diabetic retinopathy. Significant genes were further evaluated using colocalization analysis, functional enrichment, protein–protein interaction network analysis, drug prediction, and molecular docking. Finerenone was subsequently assessed in db/db mice and in the oxygen-induced retinopathy model. Thirty candidate druggable genes were associated with diabetic retinopathy after false discovery rate correction. Among them, CDH2 was the only candidate showing significant colocalization with diabetic retinopathy risk, with a posterior probability for a shared causal variant of 0.85. Protein–protein interaction analysis showed relatively high connectivity of CDH2 within the candidate network, and molecular docking suggested a favorable predicted interaction between finerenone and N-cadherin. *In vivo*, finerenone reduced avascular and neovascular areas in oxygen-induced retinopathy retinas and decreased retinal expression of TNF-α, IL-1β, and N-cadherin in db/db mice. These findings prioritize CDH2/N-cadherin as a genetically supported candidate target in diabetic retinopathy and support finerenone as a potential therapeutic candidate. The retinal protective effects of finerenone may be associated with suppression of inflammatory responses and downregulation of N-cadherin, although the direct mechanistic link requires further validation.

## Introduction

Diabetic retinopathy (DR) is one of the most common complications of diabetes and remains a leading cause of visual impairment and blindness in working-age adults (Antonetti et al., 2021; Vujosevic et al., 2020). Although improved glycaemic control since 1980 has contributed to a decline in the prevalence of all stages of DR, the global burden of visual impairment and blindness caused by DR increased between 1990 and 2015 (Vujosevic et al., 2020), and the age-standardized rate of blindness attributable to diabetic eye disease increased from 14.9% to 18.5% between 1990 and 2020 (Steinmetz et al., 2021). In 2020, more than 103 million people worldwide were affected by DR, and this number is projected to increase by 60% over the next 25 years (Teo et al., 2021).

Current treatment of DR remains challenging. Before the introduction of vascular endothelial growth factor A (VEGF-A)-targeted therapy, laser photocoagulation was the only therapeutic option, although it may cause substantial visual field loss (Arrigo et al., 2022; Zhang et al., 2012). Anti-VEGF therapy is now the first-line treatment, but it requires repeated intravitreal injections and not all patients respond adequately (Arrigo et al., 2022). Given the limitations of current therapies, a better understanding of the genetic basis of DR susceptibility is needed. The molecular basis of DR remains incompletely understood. Identifying genes involved in the development and progression of DR may facilitate the discovery of new therapeutic targets.

Mendelian randomization (MR) uses genetic variants as instrumental variables to assess the relationship between a modifiable exposure and an outcome (Sanderson et al., 2022). Based on Mendel’s laws of inheritance and the principle that gene expression represents a lifelong exposure, MR can approximate the design of a randomized controlled trial without requiring therapeutic intervention (Finan et al., 2017) and can reduce bias caused by unmeasured confounding between exposure and outcome (Burgess et al., 2015). The druggable genome refers to the subset of genes that encode proteins with potential therapeutic relevance and may therefore inform target identification and drug development (Finan et al., 2017). In the context of diabetic retinopathy, integrating these approaches may help bridge human genetic evidence and candidate drug discovery. In the present study, we performed a druggable Mendelian randomization analysis integrating druggable-gene resources, blood cis-eQTL data, and a large DR GWAS dataset to identify genetically supported therapeutic candidates. We then used colocalization analysis, network-based evaluation, and molecular docking to prioritize candidate targets, followed by experimental validation in db/db and oxygen-induced retinopathy models. This design was intended to move from human genetic target nomination to preclinical assessment of therapeutic relevance in diabetic retinopathy.

## Materials and methods

**FIGURE 1:**
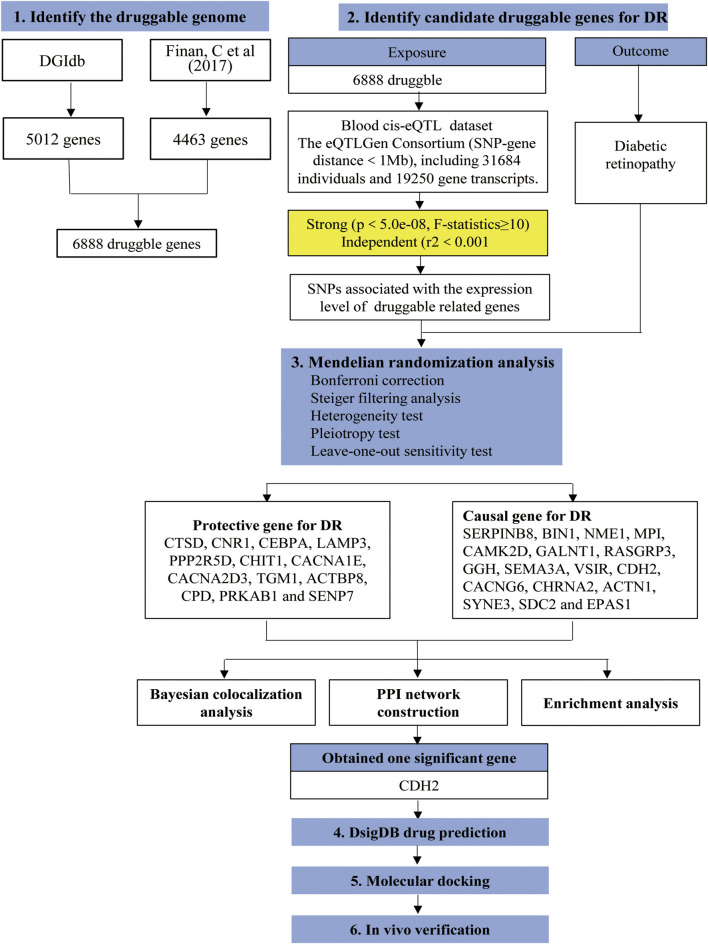
Overview of the study design. DGIdb, Drug–Gene Interaction Database; eQTL, expression quantitative trait locus; GWAS, genome-wide association study; PPI, protein–protein interaction; DSigDB, Drug Signatures Database.

The flow chart of this study is presented in Figure 1. More details of the methods and materials used are provided as follows.

We obtained druggable genes from the Drug–Gene Interaction Database (DGIdb, version 5.0, https://www.dgidb.org/). The DGIdb contains over 70,000 drug-gene interactions, involving 10,000 genes and 20,000 drugs targeting these genes (Freshour et al., 2021). It has been updated to version 5.0. We downloaded the ‘Categories Data’ of the latest version (released in June 2024), including all genes in the druggable categories of the DGIdb. We also obtained a list of druggable genes from the review from Finan et al. (Finan et al., 2017), and combined them with the DGIdb database to get a broader range of druggable genes.

### eQTL datasets

We obtained the blood eQTL data from eQTLGen (https://eqtlgen.org/), which incorporated 37 datasets (Võsa et al., 2021). The eQTLGen collected cis-eQTLs for 16,987 genes derived from 31,684 blood samples supplied by European ancestry individuals (Võsa et al., 2021). Firstly, correlation analysis was conducted by filtering data with p-value less than 5e-08 to identify SNPs strongly correlated with exposure factors. Secondly, removed SNPs with linkage disequilibrium based on kb = 10,000 and *r* ^2^ = 0.001. Lastly, F-test was performed to remove the influence of weak instrumental variables by filtering data with F-test values greater than 10.

### DR GWAS dataset

The outcome data in this study were introduced from a large-scale DR GWAS, which comprised 64,091 individuals of European ancestry included 12,681 clinically confirmed DR cases and 51,410 controls (Table 1).

**TABLE 1:** Data sources for MR analysis in the current study.

Type of dataset	Data subtype	Source	Sampel size	Population	Download site
Druggable genome	DGIdb 4.0	Freshour et al. (2020)	-	-	https://www.dgidb.org/downloads
	Prior druggable gene	Finan et al. (2017)	-	-	Finan C, et al. Pmid: 28356508
QTL datasets	Blood cis-eQTL	eQTLGen consortium (Võsa et al. 2021)	31,684	Europea	https://www.eqtlgen.org/cis-eqtls.htm
GWAS summary	Diabetic retinopathy	-	Cases: 12,681	Europea	https://storage.googleapis.com/finngen-public-data-r11/summary stats/finngen_R11_DM_RETINOPATHY_EXMORE.gz
			Controls: 51,410		

DGIdb, drug-gene interaction database; eQTL, expression quantitative trait loci; eQTLGen, Consortium, expression quantitative trait loci generation consortium; GWAS, genome-wide association study; ^a^Sample size shown as a total number for quantitative traits, continuous traits and cases/controls for binary traits.

### Mendelian randomization analysis

We conduct MR analyses using the ‘Two Sample MR’ package (version 0.5.7) in R^12^. The eQTLs of the druggable genome were introduced as the exposure data. For construction of IVs, SNPs located within±100 kb of the transcriptional start site (TSS) of each gene with a FDR less than 0.05 were picked. These SNPs were then clumped at an *r* ^2^ less than 0.001 using European samples from the 1000 Genomes Project (Auton et al., 2015). The R package ‘phenoscanner’ (version 1.0) was introduced to identify phenotypes related to the IVs. Moreover, we excluded SNPs that were directly associated with DR. The filtered SNPs were harmonized and analyzed using the ‘Two Sample MR’ package. If only one SNP was obtained for analysis, the Wald ratio method was introduced to perform MR estimation. If multiple SNPs were available, the inverse-variance weighted (IVW) method was used to perform MR analysis. Heterogeneity was assessed using Cochran’s Q test (Greco et al., 2015) and pleiotropy was evaluated via MR Egger’s intercept (Andersen et al., 2017). We conducted MR analyses using the TwoSampleMR package (version 0.5.7) in R (Hemani et al., 2018).

### Colocalization analysis

We conducted Bayesian analysis using the ‘coloc’package in R (http://cran.r-project.Org/web/packages/coloc) to investigate whether the observed association between the two traits (Trait1: potential druggable genes, Trait 2: DR-related traits) was out of the same genetic variant. Five hypotheses were supported by the following probability (Giambartolomei et al., 2014): P1, not associated with any trait; PPH1, related to gene expression but not associated with DR risk; PPH2, associated with DR risk but not related to gene expression; PPH3, associated with both DR risk and gene expression, with clear causal variation; and PPH4, associated with both DR risk and gene expression, with a common causal variant.

### Enrichment analysis

Gene Ontology (GO) analysis was performed using R package ‘clusterProfler’ (version 4.10.1) to investigate the characteristics of functional and biological relevance among the predetermined prospective druggable genes (Yu et al., 2012). GO analysis included three terms: Biological Process (BP), Cellular Component (CC) and Molecular Function (MF). Kyoto Encyclopedia of Genes and Genomes (KEGG) enrichment studies can provide information about metabolic pathways.

### Protein–protein interaction network construction

We performed protein–protein interaction (PPI) networks using the STRING (https://string-db.org/) to illustrate the relationships between protein interactions of significant druggable genes. A confidence score threshold of 0.4 was deemed as the minimum required interaction score, while all other parameters were maintained as default (Szklarczyk et al., 2023).

### Candidate drug prediction

Drug Signatures Database (DSigDB, http://dsigdb.tanlab.org/DSigDBv1.0/) is a collection of drug and small molecule-related gene sets based on quantitative inhibition and/or drug-induced gene expression changes data (Yoo et al., 2015). 22,527 gene sets consisting of 17,389 unique compounds spanning 19,531 genes were collected. We combined the significant druggable genes to DSigDB to predict candidate drugs and evaluate the pharmacological activity of target genes.

### Molecular docking

Molecular docking, a silico structure-based strategy was conducted to identify novel compounds of therapeutic interest, predict ligand-target interactions at a molecular level and delineate structure-activity relationships (SAR) (Pinzi and Rastelli, 2019). Firstly, we source the drug structure in PubChem Compound Database (https://pubchem.ncbi.nlm.nih.gov/) (Kim et al., 2023). These data were downloaded in SDF pattern and converted to pdb format using OpenBabel 2.4.1. Secondly, we downloaded protein structural data from the Protein Data Bank (PDB, http://www.rcsb.org/). Thirdly, we chose the drugs that we interested in and the proteins encoded by the colocalized gene to perform molecular docking using the AutoDock 4.2.6 (http://autodock.scripps.edu/) (Morris et al., 2009), and visualized the results by PyMol 3.0.2 (https://www.pymol.org/).

### Animal experimental design

All animal procedures were approved by the Laboratory Animal Ethical Committee of Tianjin Medical University Chu Hsien-I Memorial Hospital (approval no. DXBYY-IACUC-2023026) and were conducted in accordance with the National Institutes of Health Guide for the Care and Use of Laboratory Animals. The reporting of animal experiments in this study complies with the ARRIVE guidelines. No human participants or newly collected human biological specimens were involved in this study. Seven-week-old specific pathogen-free male db/m and db/db mice were obtained from Gempharmatech Co., Ltd. Body weights ranged from 20 to 25 g in db/m mice and from 30 to 40 g in db/db mice. All mice were housed in the institutional Laboratory Animal Center under controlled conditions, with a temperature of 23 °C ± 3 °C, relative humidity of 45% ± 15%, and a 12 h light/12 h dark cycle, and were given free access to food and water throughout the study. The db/db mice were randomly assigned to either the finerenone treatment group or the vehicle group, whereas db/m mice served as the normal control group. Six biologically independent mice were included in each of the db/m control, db/db vehicle, and db/db finerenone groups. All six animals per group were included in the analyses of body weight, fasting plasma glucose, serum triglycerides, total cholesterol, retinal qRT-PCR, and retinal Western blotting. Finerenone (BAY 94–8,862, Leverkusen, Germany) was administered by oral gavage at a dose of 3 mg/kg/day starting at 8 weeks of age and continued for 10 consecutive weeks. The vehicle and normal control groups received an equal volume of solvent by oral gavage for the same duration (0.2 mL per mouse). At the end of the treatment period, mice used for retinal tissue collection were deeply anesthetized with isoflurane inhalation. Anesthesia was induced with 4% isoflurane and maintained with 2% isoflurane in oxygen at a flow rate of 1.0 L/min. Adequate anesthesia was confirmed by the absence of pedal withdrawal and corneal reflexes. Mice were then euthanized by cervical dislocation under deep anesthesia, and death was confirmed by cessation of respiration and heartbeat before retinal tissue collection.

### Oxygen-induced retinopathy mouse model

Given that diabetic rodent models fail to develop proliferative diabetic retinopathy (PDR), we utilized the oxygen-induced retinopathy (OIR) model, a well-established experimental system characterized by robust retinal neovascularization. To establish the OIR model, male and female C57BL/6J mouse pups at postnatal day 7 (P7), together with their nursing dams, were placed in a regulated hyperoxic chamber (75% ± 2% O_2_) until P12. Oxygen concentration was continuously monitored and maintained within the target range. The pups were then returned to room air (normoxia, 21% O_2_) and maintained under room air (21% O_2_) until P17. Age-matched pups maintained under normoxia throughout the experimental period served as the normoxia control group. OIR mice received either vehicle (0.9% NaCl; OIR + vehicle) or finerenone (3 mg/kg/day; OIR + finerenone) once daily from P12 to P16 (for 5 consecutive days). Vehicle was administered at an equal volume to the finerenone group. All retinal tissues were collected at P17 for subsequent assays, and comparisons were performed among the normoxia, OIR + vehicle, and OIR + finerenone groups. Six biologically independent mice were included in each of the NOIR, OIR + vehicle, and OIR + finerenone groups for the retinal flat-mount analysis.

### Cell culture and treatments

Human retinal microvascular endothelial cells (HRMECs; BNCC, China) were cultured in Endothelial Cell Medium (ECM; ScienCell, USA) supplemented with 5% fetal bovine serum. Cells were maintained at 37 °C in a humidified atmosphere containing 5% CO_2_. HRMECs were assigned to three groups: normal glucose (NG; 5.5 mmol/L glucose), high glucose (HG; glucose was added to achieve a final concentration of 25 mmol/L), and high glucose plus finerenone (HG + finerenone; 25 mmol/L glucose and 10 μmol/L finerenone). Finerenone (HY-111372; MedChemExpress, USA) and high glucose were administered simultaneously for 48 h. Equivalent volumes of DMSO were added to the NG and HG groups as vehicle controls. Three independent biological experiments were performed.

### Fluorescein isothiocyanate (FITC)-conjugated dextran staining of whole-mount retinas

At P17, OIR mice were deeply anesthetized with isoflurane inhalation before cardiac exposure. Anesthesia was induced with 4% isoflurane and maintained with 2% isoflurane in oxygen at a flow rate of 1.0 L/min. Adequate anesthesia was confirmed by the absence of pedal withdrawal and corneal reflexes. A solution of FITC-dextran (50 mg/mL, Sigma-Aldrich, Cat. No. FD 2000S) was then injected into the left ventricular cavity at a volume of 200 μL per mouse. After 5 min of circulation, mice were euthanized by cervical dislocation under deep anesthesia, and eyeballs were immediately enucleated and immersed in 4% paraformaldehyde. Retinal flat-mounts were prepared via careful dissection, followed by image acquisition under a laser scanning confocal microscope. This FITC-dextran labeling approach enabled visualization of the retinal vascular network, thus facilitating quantitative evaluation of both the avascular retinal regions and neovascular tuft formation.

### Quantitative real-time polymerase chain reaction (qRT-PCR)

Total RNA was extracted from retinal tissues using TRIzol reagent and reverse-transcribed into complementary DNA (cDNA), with RNA quality verified by OD_260_/OD_280_ ratio (1.8–2.0). qRT-PCR was performed on an ABI 7500 system using SYBR Green Premix. Primers for tumor necrosis factor-α (TNF-α), interleukin-1β (IL-1β), neural cadherin (Ncad), and 18S rRNA were synthesized by Sangon Biotech (primer sequences are provided in Supplementary Table S3). The 20 μL reaction system contained 10 μL Premix, 0.4 μL each primer, 2 μL cDNA and 7.2 μL RNase-free water. Amplification conditions were as follows: 95 °C for 3 min; 40 cycles of 95 °C for 10 s, 60 °C for 30 s, 72 °C for 20 s; followed by melting curve analysis included. Relative mRNA expression was calculated using the 2^-ΔΔCt method with 18S rRNA as the internal control, and all samples were analyzed in technical triplicates.

### Western blotting

Retinal tissues and HRMECs were lysed on ice in RIPA lysis buffer (Solarbio, China) supplemented with protease inhibitors. Equal amounts of protein were separated by sodium dodecyl sulfate–polyacrylamide gel electrophoresis and transferred onto nitrocellulose membranes. After blocking with 5% skim milk for 1 h at room temperature, the membranes were incubated overnight at 4 °C with primary antibodies against IL-1β (1:1,000; Abcam, Cat. No. ab234437), TNF-α (1:1,000; Proteintech, Cat. No. 17590-1-AP), N-cadherin (1:1,000; Cell Signaling Technology, Cat. No. 13116), VEGF (1:1,000; Proteintech, Cat. No. 19003-1-AP), and Tubulin (1:8,000; Zen-Bioscience, Cat. No. R380628). The membranes were subsequently incubated with horseradish peroxidase-conjugated anti-rabbit or anti-mouse secondary antibodies (1:5,000; Sungene Biotech) for 1 h at room temperature. Immunoreactive bands were visualized using an enhanced chemiluminescence kit (WBKLS0500; Millipore) and quantified using ImageJ software (version 1.54r; National Institutes of Health, Bethesda, MD, USA). Target protein levels were normalized to Tubulin. HRMEC Western blot analyses were performed in three independent biological experiments. For the OIR retinal Western blot analyses, six biologically independent mice were included in each group.

### Immunofluorescence staining

Paraffin-embedded retinal sections from the NOIR, OIR + vehicle, and OIR + finerenone groups were deparaffinized, rehydrated, and subjected to heat-induced antigen retrieval. After blocking with 10% goat serum containing 0.1% Triton X-100 for 1 h at room temperature, the sections were incubated overnight at 4 °C with primary antibodies against CD31 (1:50; Thermo Fisher Scientific, Cat. No. MA1-26196) and N-cadherin (1:50; Proteintech, Cat. No. 22018-1-AP). After washing, the sections were incubated with species-appropriate FITC- and TRITC-conjugated secondary antibodies (1:200; Simubiotech, China) for 2 h at room temperature in the dark. Nuclei were counterstained with DAPI. Images were acquired using an Olympus BX53 microscope (Olympus, Tokyo, Japan) under identical acquisition settings across all experimental groups. Retinal sections from six biologically independent mice per group were examined, and representative images are presented.

### Statistical analysis

Statistical analyses were performed using GraphPad Prism 10.6.1 software (GraphPad, RRID:SCR_000306) (Curtis et al., 2018). Normal probability plots were employed to verify the normality of data distributions, with experimental results expressed as the mean ± standard deviation. Student’s t-test was applied for pairwise group comparisons, whereas one-way ANOVA combined with Tukey’s or Sidak’s multiple comparison corrections was used for multi-group analyses. Statistical significance was set at *P* < 0.05.

## Results

### Druggable genome

5,012 druggable genes from the DGIdb and 4,463 druggable genes from the abovementioned review (Finan et al., 2017) were integrated. 6,888 unique druggable genes were obtained for the following analysis.

### Candidate druggable genes

**FIGURE 2:**
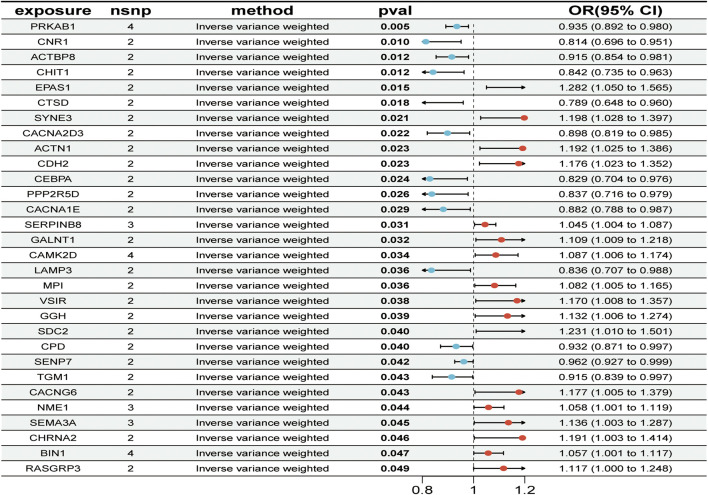
Candidate druggable genes associated with diabetic retinopathy identified using the Wald ratio or inverse-variance weighted method.

After removing SNPs beyond±100 kb from the TSS, 6,959 SNPs associated with 3,846 druggable gene symbols were obtained to perform MR analysis. After ruling out the SNPs directly related to DR via PhenoScanner, 30 candidate druggable genes for a potential causal relationship with DR were identified following FDR adjustment (FDR <0.05) using the Wald ratio or the IVW method (Figure 2). Detailed results for the significant IVs and full results of MR are available in the Supplementary Table S1. A full suite of sensitivity analyses including leave-one-out MR, MR-Egger regression, and MR-PRESSO tests were performed to validate the robustness of our observed associations. These analyses yielded no evidence of horizontal pleiotropy or influential outliers, supporting the robustness of the observed genetic associations. Among the 30 potential druggable genes, 13 genes are recognized as protective factors (CTSD, CNR1, CEBPA, LAMP3, PPP2R5D, CHIT1, CACNA1E, CACNA2D3, TGM1, ACTBP8, CPD, PRKAB1 and SENP7) and 17 genes are identified as risk factors (SERPINB8, BIN1, NME1, MPI, CAMK2D, GALNT1, RASGRP3, GGH, SEMA3A, VSIR, CDH2, CACNG6, CHRNA2, ACTN1, SYNE3, SDC2 and EPAS1).

### Enrichment analysis

**FIGURE 3:**
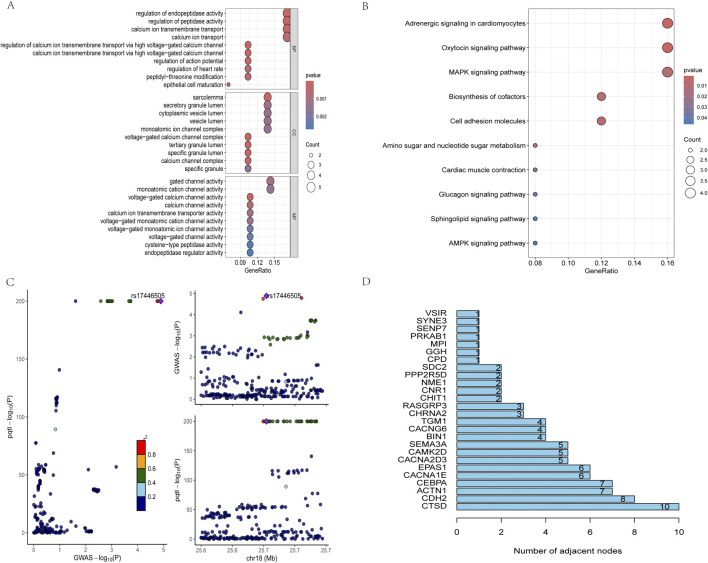
Analysis of the 30 candidate druggable genes. **(A)** GO enrichment analysis. **(B)** KEGG enrichment analysis. **(C)** Colocalization analysis using Bayesian analysis. **(D)** PPI network analysis using the STRING database.

GO analysis of 30 potential targets showed that these targets are primarily involved in BP such as regulation of endopeptidase activity (GO:0052548), regulation of peptidase activity (GO:0052547), calcium ion transmembrane transport (GO:0070588), and calcium ion transport (GO:0006816). The main CC include sarcolemma (GO:0042383), secretory granule lumen (GO:0034774), cytoplasmic vesicle lumen (GO:0060205), vesicle lumen (GO:0031983) and monoatomic ion channel complex (GO:0034702). The main MF include gated channel activity (GO:0022836) and monoatomic cation channel activity (GO:0005261) (Figure 3A). KEGG analysis showed that the target genes were primarily enriched in pathways such as Adrenergic signaling in cardiomyocytes, Oxytocin signaling pathway and MAPK signaling pathway (Figure 3B).

### Colocalization analysis

A posterior probability for hypothesis 4 (PPH4) > 0.75 was considered evidence consistent with colocalization between gene expression and DR risk. Among the 30 candidate genes, only CDH2 exceeded this threshold, with a PPH4 of 0.85, indicating that the two association signals were compatible with a shared causal variant (Figure 3C).

### Protein–protein interaction network construction

We constructed a PPI network by uploading the 30 candidate druggable genes to the STRING database. The resulting network consisted of 26 nodes and 47 edges. CTSD showed the highest connectivity, with connections to 10 other candidate proteins, whereas CDH2 was connected to eight other candidate proteins, indicating relatively high connectivity within the candidate network (Figure 3D).

### Candidate drug prediction

**FIGURE 4:**
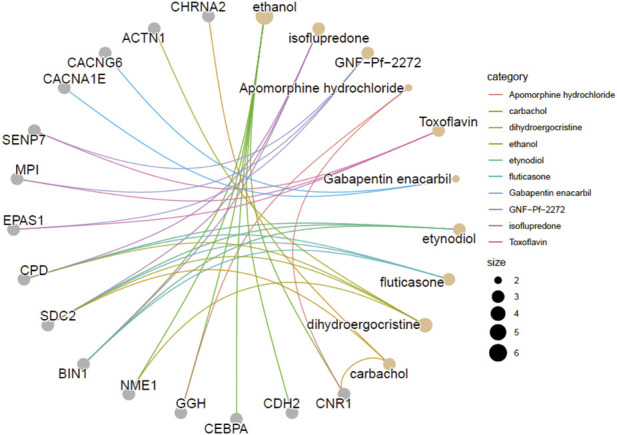
Gene–drug interaction network showing interactions between significant genes and predicted therapeutic compounds.

Drug-associated gene sets were queried using DSigDB, and the top 10 compounds were ranked according to their adjusted P values (Table 2). Ethanol and isoflupredone were the two highest-ranked compounds identified in this analysis. Ethanol was associated with a gene signature containing CNR1, CDH2, CEBPA, GGH, NME1, BIN1, and SDC2, whereas isoflupredone was associated with CPD and BIN1. To further investigate these relationships, a gene-drug interaction network was established, offering a holistic overview of the interactions between the significant genes and potential therapeutic compounds (Figure 4). According to the colocalization analysis, CDH2 was the only candidate showing evidence consistent with a shared causal variant with DR. In addition, CDH2 showed relatively high connectivity within the candidate network, with associations involving eight of the other candidate proteins. Based on the combined colocalization and network findings, CDH2 was prioritized for exploratory drug-association analysis. Except for the ethanol (obtained from the drug enrichment analysis), we identified 170 compounds associated with CDH2-containing drug signatures in DSigDB (Supplementary Table S2). Of these, aldosterone was selected as a candidate molecule.

**TABLE 2:** Candidate drug predicted by DsigDB.

Drug name	*p* value	*p*.Adjust	Gene ID	Count
Ethanol	1.62E-05	0.009073	CNR1/CDH2/CEBPA/GGH/NME1/BIN1	6
Isoflupredone	0.000262	0.03466	SDC2/CPD/BIN1	3
GNF-pf-2272	0.000329	0.03466	EPAS1/MPI/SENP7	3
Apomorphine hydrochloride	0.000366	0.03466	CNR1/GGH	2
Toxoflavin	0.000371	0.03466	EPAS1/MPI/SENP7	3
Gabapentin enacarbil	0.000486	0.03466	CACNA1E/CACNG6	2
Etynodiol	0.000532	0.03466	SDC2/CPD/BIN1	3
Fluticasone	0.000561	0.03466	SDC2/CPD/BIN1	3
Dihydroergocristine	0.000582	0.03466	ACTN1/SDC2/CPD/NME1	4
Carbachol	0.00062	0.03466	CNR1/SDC2/CHRNA2	3

### Molecular docking

**FIGURE 5:**
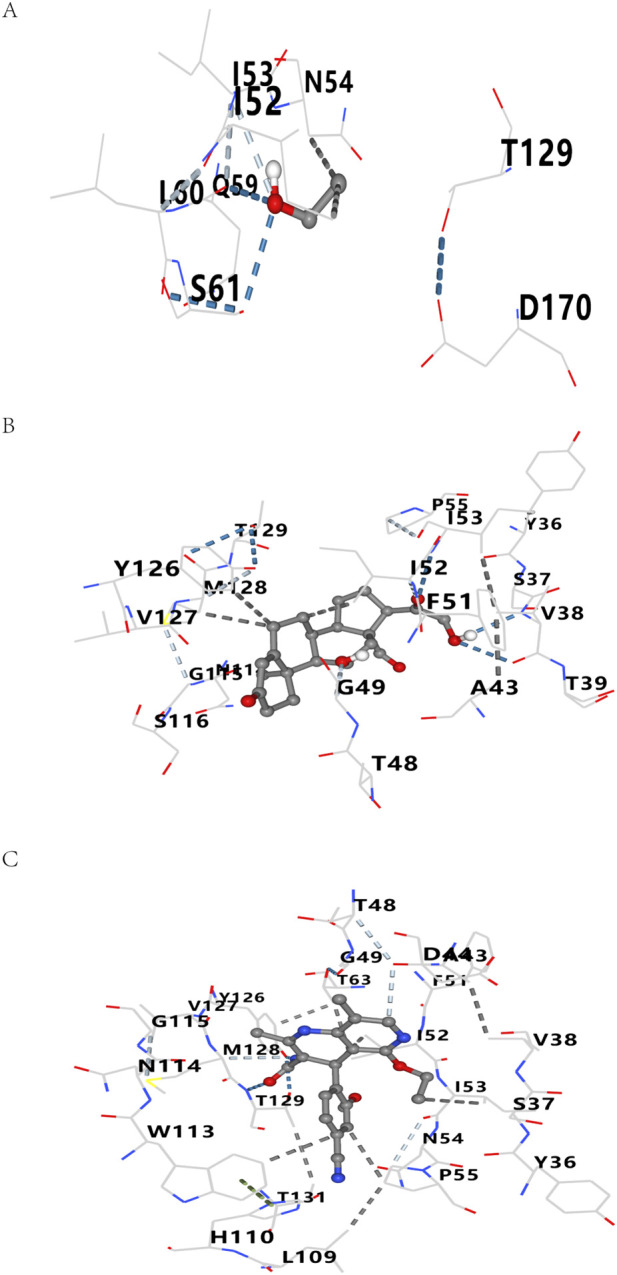
Predicted structure-based docking of N-cadherin with ethanol, aldosterone, and finerenone. **(A)** Predicted docking of ethanol with N-cadherin. **(B)** Predicted docking of aldosterone with N-cadherin. **(C)** Predicted docking of finerenone with N-cadherin.

We used CB-Dock2 to explore the predicted docking interactions between the selected compounds and N-cadherin, the protein encoded by CDH2. Aldosterone yielded a Vina score of −7.6 kcal/mol, indicating a favorable predicted docking interaction. Finerenone yielded a Vina score of −7.7 kcal/mol. The predicted binding poses, interacting amino acid residues, and hydrogen-bond distances are shown in Figure 5. These computational results were used for hypothesis generation and do not establish direct target binding.

### *In vivo* verification

**FIGURE 6:**
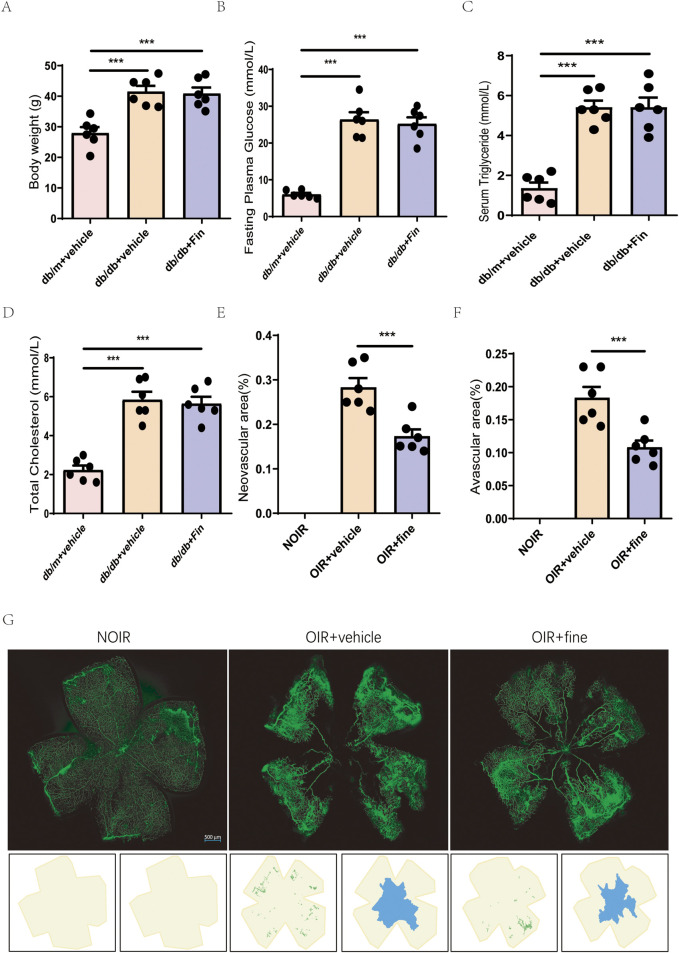
**(A–D)** Body weight, fasting plasma glucose, serum triglyceride, and total cholesterol levels in db/m, db/db, and finerenone-treated db/db mice (n = 6 biologically independent mice per group). **(E–G)** Representative retinal flat-mounts and quantification of avascular and neovascular areas in NOIR, OIR + vehicle, and OIR + finerenone mice (n = 6 biologically independent mice per group). Each data point represents one animal.

**FIGURE 7:**
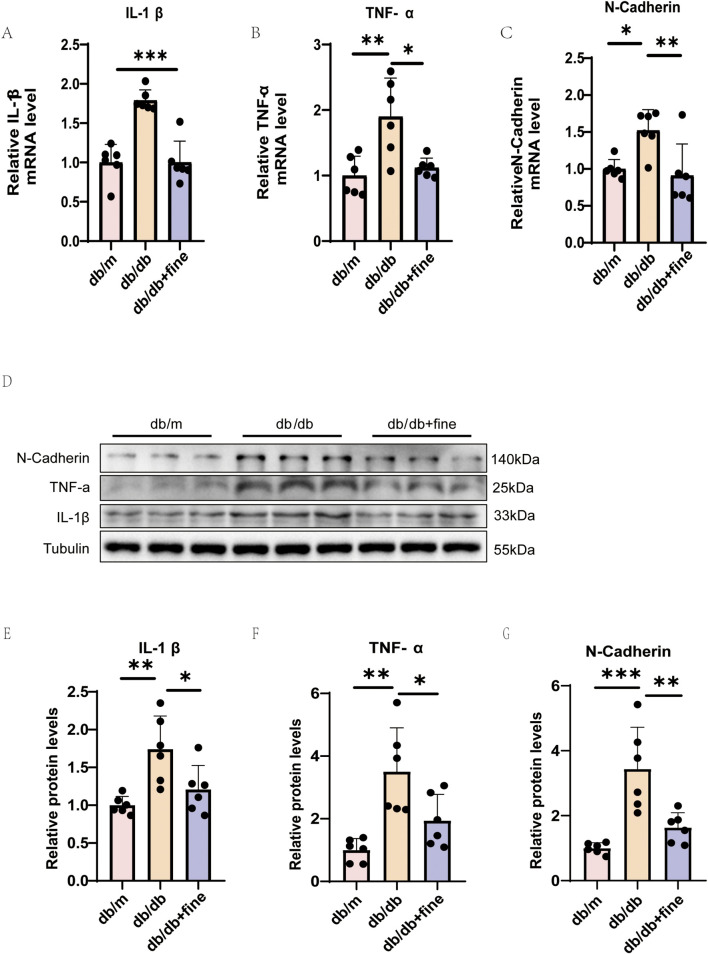
Finerenone decreased retinal inflammatory markers and N-cadherin expression in db/db mice. **(A)** Relative IL-1β mRNA expression. **(B)** Relative TNF-α mRNA expression. **(C)** Relative N-cadherin mRNA expression. **(D)** Representative Western blot bands for N-cadherin, TNF-α, IL-1β, and Tubulin in db/m, db/db, and db/db + finerenone groups. **(E)** Quantification of IL-1β protein expression. **(F)** Quantification of TNF-α protein expression. **(G)** Quantification of N-cadherin protein expression. Data are presented as mean ± SD. *P < 0.05, **P < 0.01, and ***P < 0.001.

To validate the MR findings and evaluate Finerenone-mediated retinal benefits in db/db mice, the aforementioned oral administration protocol was adopted. Finerenone was administered once daily via oral gavage for a consecutive 10-week period, commencing when the mice reached 8 weeks of age. No statistically significant changes in body weight, FPG, serum triglyceride, or total cholesterol levels were observed in the finerenone-treated group (Figures 6A–D). We also investigated the changes in avascular area and neovascular area between the OIR mice treated with or without Finerenone. Compared with the NOIR group, the OIR + vehicle group showed significantly increased avascular and neovascular areas, whereas finerenone treatment significantly reduced both areas (Figures 6E–G). Since the anti-inflammatory and anti-fibrotic effects of Finerenone in DKD have been confirmed in multiple studies, we investigated the expression of pro-inflammatory mediators in the retinas of db/db mice. We found that compared with db/m mice, the mRNA of TNF-α and IL-1β increased significantly in the retinas of db/db mice, and finerenone reduced the mRNA level of the two pro-inflammatory factors. Meanwhile, we also detected the mRNA of Ncad which was coded by CDH2. We found that the mRNA of Ncad in db/db mice was increased, and in the finerenone-treated group, Ncad was decreased (Figure 7A). These changes were confirmed by Western blot in protein level (Figures 7B,C).

### Finerenone attenuates N-cadherin, inflammatory mediators, and VEGF expression in OIR retinas and HRMECs

**FIGURE 8:**
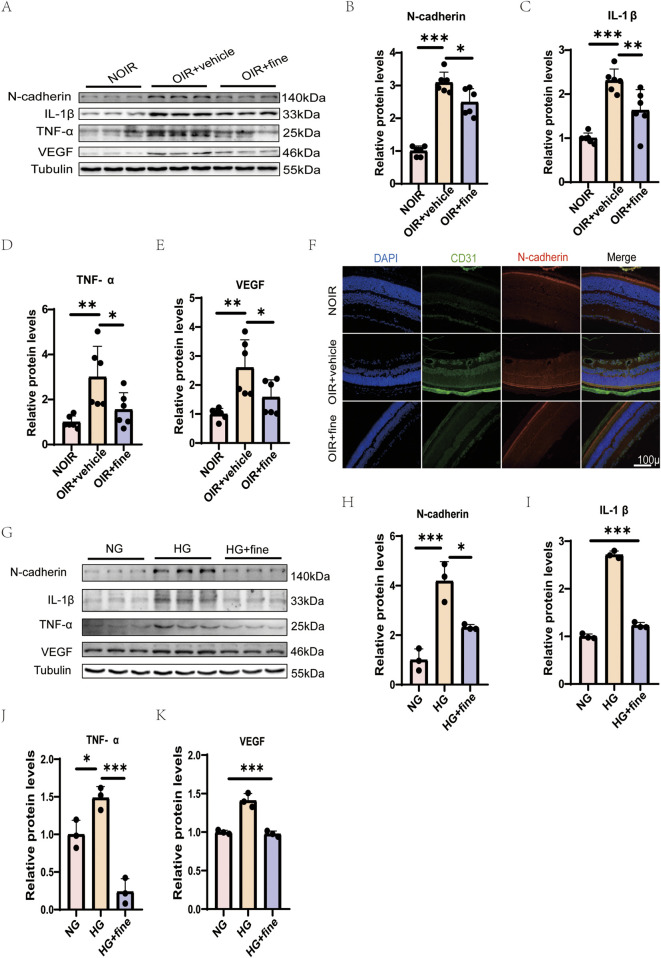
Finerenone attenuates N-cadherin, inflammatory mediators, and VEGF expression in OIR retinas and high-glucose-exposed HRMECs. **(A)** Representative immunoblots of N-cadherin, IL-1β, TNF-α, VEGF, and Tubulin in retinas from the NOIR, OIR + vehicle, and OIR + finerenone groups. **(B–E)** Quantitative analysis of retinal N-cadherin, IL-1β, TNF-α, and VEGF protein expression, respectively. Protein expression was normalized to Tubulin (n = 6 biologically independent mice per group), **(F)** Representative immunofluorescence images of retinal sections stained for CD31 (green), N-cadherin (red), and DAPI (blue) in the NOIR, OIR + vehicle, and OIR + finerenone groups. Representative images were obtained from six biologically independent mice per group. Scale bar, 100 μm. **(G)** Representative immunoblots of N-cadherin, IL-1β, TNF-α, VEGF, and Tubulin in HRMECs exposed to normal glucose, high glucose, or high glucose plus finerenone. **(H–K)** Quantitative analysis of N-cadherin, IL-1β, TNF-α, and VEGF protein expression in HRMECs, respectively. Protein expression was normalized to Tubulin, and data were obtained from three independent biological experiments (n = 3). Data are presented as the mean ± SD. Statistical significance was assessed using one-way ANOVA followed by Tukey’s multiple-comparisons test. *P < 0.05, **P < 0.01, and ***P < 0.001.

To provide additional retinal and cellular molecular evidence, we examined N-cadherin, IL-1β, TNF-α, and VEGF expression in OIR retinas and high-glucose-exposed HRMECs. In OIR retinas, N-cadherin, IL-1β, TNF-α, and VEGF protein expression was significantly increased in the OIR + vehicle group compared with the NOIR group. Finerenone treatment significantly reduced the expression of these proteins compared with the OIR + vehicle group (Figures 8A–E). Representative immunofluorescence images showed more prominent CD31 and N-cadherin staining in OIR + vehicle retinas than in NOIR retinas, with visibly attenuated staining following finerenone treatment (Figure 8F).

Consistent changes were observed in HRMECs. High-glucose exposure significantly increased N-cadherin, IL-1β, TNF-α, and VEGF protein expression compared with the normal-glucose group, whereas finerenone treatment significantly reduced the expression of these proteins compared with the high-glucose group (Figures 8G–K). Together, these findings provide additional retinal and cellular evidence that finerenone treatment is associated with reduced N-cadherin expression and attenuation of inflammatory and angiogenesis-related molecular changes.

## Discussion

Druggable Mendelian randomization provides a useful framework for identifying genetically supported repurposing opportunities for licensed drugs (Finan et al., 2017). In this study, we integrated human genetic target prioritization with experimental validation to investigate therapeutic opportunities for diabetic retinopathy. Among the 30 significant druggable genes identified by Mendelian randomization, CDH2 was the only gene showing significant colocalization evidence with DR risk and therefore had the strongest combined genetic support among the candidates evaluated in this study. Protein–protein interaction analysis further showed relatively high connectivity of CDH2 within the candidate network. On this basis, we evaluated finerenone as a candidate therapeutic intervention and found that finerenone reduced pathological retinal neovascularization in the OIR model and attenuated retinal inflammatory responses in db/db mice.

This framework is potentially relevant from a translational perspective. The mineralocorticoid receptor is an established therapeutic target in CKD (Agarwal et al., 2022), and finerenone has been shown to slow CKD progression and reduce cardiovascular risk in patients with CKD and T2D in the phase 3 FIDELIO-DKD and FIGARO-DKD trials (Bakris et al., 2020; Pitt et al., 2021). Further mechanistic studies have demonstrated additional anti-inflammatory and anti-fibrotic effects of MR antagonism beyond its classical renal actions, such as blood pressure regulation and maintenance of salt and water homeostasis (Barrera-Chimal et al., 2022). Over the past 2 decades, multiple studies have shown that mineralocorticoid receptor activation exerts cell-specific effects in cardiomyocytes, endothelial cells, vascular smooth muscle cells, adipocytes, and inflammatory cells (Jaisser and Farman, 2016; Barrera-Chimal et al., 2019). Given that the mineralocorticoid receptor is overexpressed in the retina of type 2 diabetic Goto-Kakizaki rats and in humans, it is reasonable to hypothesize that finerenone may exert protective effects in DR (Zh et al., 2021). A pooled analysis of the ReFineDR and DeFineDR studies suggested that finerenone may delay the progression of NPDR to vision-threatening complications and reduce the need for ocular interventions (Rossing et al., 2023). Jerome and colleagues compared finerenone with the angiotensin-converting enzyme inhibitor perindopril in diabetic and hypertensive transgenic (mRen-2) rats and found that finerenone reduced neovascularization, vascular leakage, and microglial density, increased Treg levels in the blood, spleen, and retina, and decreased retinal VEGF, ICAM-1, and IL-1β expression (Jerome et al., 2023). Our findings extend this concept by linking human genetic target prioritization to preclinical retinal validation, thereby supporting finerenone as a candidate therapeutic strategy in DR, although the mechanisms underlying its retinal protective effects remain incompletely understood.

Notably, finerenone treatment did not significantly alter body weight, fasting plasma glucose, serum triglyceride, or total cholesterol levels in db/db mice. This finding is pharmacologically plausible because finerenone is a selective non-steroidal mineralocorticoid receptor antagonist rather than a glucose- or lipid-lowering agent. The retinal benefits observed in the absence of measurable systemic metabolic improvement therefore suggest that these effects were not primarily mediated by correction of hyperglycaemia or dyslipidaemia. Potential mechanisms may include suppression of mineralocorticoid receptor-dependent inflammatory and oxidative stress responses, stabilization of retinal endothelial function and the blood–retinal barrier, attenuation of VEGF-associated angiogenic signalling, and broader protection of the retinal neurovascular unit. However, because finerenone was administered orally, indirect systemic effects, including haemodynamic, renal, or immune modulation, cannot be excluded. Further studies using retina-specific or cell-specific approaches are required to distinguish direct retinal actions from secondary systemic effects.

In this study, we identified 30 druggable genes that may influence DR risk. GO analysis showed that these target genes converged on ion transport, ion channel complex or gated channel activity. These findings suggest that ion transport-related processes may be involved in DR pathogenesis. We then performed colocalization analysis, in which CDH2 was the only candidate showing significant evidence consistent with a shared causal variant with DR. Protein–protein interaction analysis further showed relatively high connectivity of CDH2 within the candidate network. CDH2 is a member of the classic cadherin family of Ca2+-dependent cell adhesion molecules. The protein encoded by CDH2, N-cadherin (Ncad), is a transmembrane glycoprotein that binds calcium ions through its extracellular domain and is involved in tumour epithelial-mesenchymal transition (Quan et al., 2021), development (Na et al., 2020), and cardiovascular biology (Yan et al., 2020) through its roles in cell adhesion and β-catenin signalling. Multiple studies have identified CDH2 as an important regulator of neurodevelopment and neurological disease (László and Lele, 2022). CDH2-based adherens junctions maintain neuroepithelial integrity in amniotes (Gänzler-Odenthal and Redies, 1998) and are involved in neurulation, neural crest development (Shoval et al., 2007), radial glial function (László et al., 2020), and neuronal differentiation during cortical development (Martinez-Garay, 2020). In the last few years, accumulating results showed the undesirable function of CDH2 in acute leukemia, glioblastoma and other solid tumors. In niche-primed leukaemia, CDH2 upregulation is associated with increased proliferation and treatment resistance (Pal et al., 2022), whereas CDH2 knockdown reduces proliferation and increases sensitivity to dexamethasone (Borbaran-Bravo et al., 2022). In glioblastoma, Ncad-mediated cell-cell adhesion contributes to adaptive radioresistance by trapping β-catenin at the cell membrane and preventing its nuclear translocation (Osuka et al., 2021). However, only a few studies have reported the role of CDH2 in DR.

DR has traditionally been considered a microvascular disease, yet increasing evidence indicates that retinal neurodegeneration is also an important component of disease progression (Wong et al., 2016). Given the broad roles of CDH2 in neurodevelopment and neurodegeneration, it is reasonable to postulate that CDH2 or Ncad may contribute to DR pathogenesis. A study from Choi Se Hyun et al. confirmed the existence of Ncad in intraretinal and neovascular tufts (Choi et al., 2018). Another study by Xiang Wei et al. reported increased Ncad expression and a positive correlation between Ncad and connective tissue growth factor (CTGF) in patients with PDR and in STZ-induced diabetic mice (Xiang et al., 2023). To our knowledge, the present study provides evidence linking retinal N-cadherin expression to inflammatory changes in experimental DR. Preclinical evidence indicates that mineralocorticoid receptor activation promotes retinal vasculopathic changes and inflammatory responses, whereas MR antagonists such as spironolactone and eplerenone attenuate blood-retinal barrier disruption, retinal neovascularization, and pro-inflammatory processes; however, the underlying mechanisms remain incompletely understood. Jerome et al. reported that finerenone suppressed VEGF, ICAM-1, and IL-1β expression and, in the OIR model, attenuated retinal neovascularization, vascular permeability, and microglial infiltration while increasing Treg levels in the blood, spleen, and retina (Jerome et al., 2023). In this study, CDH2 emerged as a genetically prioritized candidate, while finerenone treatment was accompanied by reduced N-cadherin expression and attenuation of retinal inflammatory and angiogenesis-related changes. These findings should not be interpreted as demonstrating that CDH2 is a direct molecular target of finerenone. Finerenone is an established selective non-steroidal mineralocorticoid receptor antagonist, and its retinal effects may arise through canonical mineralocorticoid receptor-dependent anti-inflammatory, antioxidant, endothelial, immune, or haemodynamic pathways that are independent of CDH2. The DSigDB and molecular docking analyses performed in this study are hypothesis-generating and do not establish direct target engagement. In parallel, CDH2/N-cadherin has broad physiological roles in neural development, vascular integrity, intercellular adhesion, and tissue homeostasis. Therefore, direct modulation of the CDH2/N-cadherin axis could have biological consequences beyond the retina, and the tissue specificity and safety of such an approach require further evaluation. The present data support an association between finerenone treatment and reduced N-cadherin expression but do not establish a CDH2-dependent mechanism.

This study has several strengths. By combining druggable Mendelian randomization with colocalization analysis, we reduced the likelihood of prioritizing targets supported only by association signals without shared genetic architecture. Network-based analysis provided additional context for candidate selection, and the *in vivo* experiments offered preclinical support for the biological relevance of the prioritized therapeutic hypothesis. More importantly, the overall design linked human genetic evidence with experimental retinal phenotypes, which strengthens the translational relevance of the study.

Several limitations should also be acknowledged. First, the Mendelian randomization analyses relied on a limited number of instrumental variables for many genes, which may affect the stability of causal estimates. Second, the genetic analyses were based on blood cis-eQTL data and European-ancestry datasets, which may not fully capture retina-specific regulation or generalize to other populations. Third, although CDH2 was genetically prioritized and finerenone treatment was accompanied by reduced N-cadherin expression, the present study did not include CDH2 knockdown or overexpression, rescue experiments, biochemical binding assays, or direct target-engagement studies. The DSigDB and molecular docking results are computational and hypothesis-generating and therefore cannot establish direct binding between finerenone and N-cadherin. Accordingly, the present data do not demonstrate that CDH2 is the direct molecular target of finerenone or that CDH2 is required for its retinal protective effects. We also did not perform systematic profiling of CDH2-independent effects of finerenone or evaluate the consequences of CDH2 modulation in non-retinal tissues. Future studies using direct CDH2 manipulation, rescue designs, and cell- or tissue-specific approaches will be required to establish causality and assess potential broader biological effects. Fourth, only male db/m and db/db mice were included in the diabetic mouse experiments. Sex-related biological differences may influence diabetic retinopathy progression and the response to finerenone; therefore, the findings obtained in the diabetic mouse model may not be fully generalizable to female animals. Both male and female pups were included in the OIR experiments, although sex-specific treatment effects were not evaluated. Future studies should include female diabetic mice and adequately powered analyses designed to evaluate potential sex-specific responses. Fifth, the db/db and OIR models were used to capture complementary aspects of disease biology, but neither model fully recapitulates the entire spectrum of human diabetic retinopathy. Further mechanistic studies, including retinal cell-specific validation and direct interrogation of the CDH2/N-cadherin axis, will be required to define the therapeutic relevance of this pathway more precisely.

## Conclusion

Through an integrated approach combining druggable Mendelian randomization with experimental validation, this study prioritized CDH2/N-cadherin as a genetically supported candidate target in diabetic retinopathy and supported finerenone as a candidate therapeutic strategy. Finerenone treatment was associated with reduced pathological retinal neovascularization, attenuation of inflammatory responses, and reduced retinal N-cadherin expression in experimental retinopathy. These findings provide a rationale for further mechanistic and translational studies of the CDH2/N-cadherin axis and finerenone in diabetic retinopathy.

## Data Availability

The datasets presented in this study can be found in online repositories. The names of the repository/repositories and accession number(s) can be found in the article/Supplementary Material.
