# Supplementary material for: Druggable Mendelian randomization prioritizes CDH2 and supports finerenone as a candidate therapeutic strategy for diabetic retinopathy
Source: Front Pharmacol. 2026 Jul 15;17:1865172. doi: 10.3389/fphar.2026.1865172 (PMC13414259; doi:10.3389/fphar.2026.1865172)
Supplement: Supplementary file 2 [file Table3.docx]

Supplementary Table 3. Primer sequences used for qRT-PCR

|  |  |  |
| --- | --- | --- |
| Gene | Forward primer (5'→3') | Reverse primer (5'→3') |
| mus-IL-1β | TGGACCTTCCAGGATGAGGACA | GTTCATCTCGGAGCCTGTAGTG |
| mus-TNF-α | GGTGCCTATGTCTCAGCCTCTT | GCCATAGAACTGATGAGAGGGAG |
| mus- Ncad | TGTTACCAGCTCGCTCTCAT | TTTCACCAGAAGCCTCCACAG |
| mus- 18S | AGGCCCTGTAATTGGAATGAGTC | GCTCCCAAGATCCAACTACGAG |
